# Momentary solitude and affective experiences: The moderating role of trait mindfulness

**DOI:** 10.1111/aphw.70052

**Published:** 2025-06-12

**Authors:** Xinyuan Peng, Da Jiang, Dwight C. K. Tse, Yuen Wan Ho, Jennifer Lay

**Affiliations:** ^1^ Department of Special Education and Counselling Education University of Hong Kong Hong Kong, China; ^2^ Department of Psychological Sciences and Health University of Strathclyde Glasgow Scotland, UK; ^3^ Department of Psychology Lingnan University Hong Kong, China; ^4^ Department of Psychology University of Exeter Exeter UK

**Keywords:** affective experiences, experience sampling method, mindfulness, solitude

## Abstract

Adults spend a significant amount of time alone every day, but mindfulness factors influencing the experience of solitude (time spent alone) are underexplored. Based on person‐environment fit theory, we examined the moderating role of five facets of trait mindfulness (*observing*, *describing*, *acting with awareness*, *nonjudging of inner experiences*, and *nonreactivity to inner experiences*) on the relationship between solitude and affective experiences. In this experience sampling study, 188 adults (*M*_*age*_ = 51.47, *SD*_*age*_ = 17.75, range = 19–93, 66.50% female) in Hong Kong completed a trait mindfulness questionnaire and then were notified five times a day for seven consecutive days to report their momentary social situation (in solitude or not) and affective states. We found that higher scores on *describing* and *acting with awareness* weakened the negative association between solitude and high‐arousal positive affect. Higher *nonreactivity to inner experiences* amplified the positive association between solitude and low‐arousal positive affect. Higher *observing* weakened the positive association between solitude and low‐arousal negative affect. These findings enhance our understanding of solitude experiences by highlighting the role of trait mindfulness in enhancing affective experiences in solitude.

## INTRODUCTION

Individuals spend a significant portion of their time in solitude (defined here as being physically alone), with a global survey indicating that people spend about 33% of their time alone each day (Statista, 2021). Time in solitude varies around this average rate, depending on age and culture. Compared to working‐age adults, who spend an estimated 30% to 40% of their time in solitude daily (e.g., Larson, 1990; Papp et al., 2013), older adults reported spending a higher proportion of their daily lives in solitude. Previous studies have shown that the percentage of time spent in solitude ranges from 48% in young adults (age 55 to 75 years; Larson et al., 1985) to 71% in the oldest old (age over 84 years old according to Chui et al., 2014). Occupying a significant amount of time in our daily routines, solitude cuts both ways. Individuals can have both positive and negative experiences in solitude (Long et al., 2003; Pauly et al., 2017). Negative experiences (for example, loneliness), in the long term, are related to a series of physical health problems (such as higher cardiovascular health risk and higher incidence of coronary heart disease) and mental health problems (such as decreased executive function and increased depressive symptoms; Hawkley & Cacioppo, 2010), while positive experiences have the potential to be nourishing and revitalizing (Long & Averill, 2003). Past research examined whether individuals with different traits experience solitude differently (e.g., Jiang et al., 2019; Zheng et al., 2023). Most of this work focuses on factors related to interpersonal traits (e.g., extraversion) or interpersonal interactions (e.g., social relationship quality and social support), defining solitude as being either physically alone or without in‐person social interaction (e.g., Birditt et al., 2019; Burger, 1995; Fang et al., 2022; Zheng et al., 2023). Solitude offers an opportunity for self‐understanding and self‐reflection, as individuals are relatively free from social demands or constraints (Jiang et al., 2019; Long & Averill, 2003). Given that key facets of trait mindfulness, such as nonjudgmental awareness and attention to oneself and the present moment are associated with increased well‐being (e.g., Kabat‐Zinn, 1990; Ortet et al., 2020; Roemer et al., 2021), we examined the moderating role of different facets of trait mindfulness in the relationship between solitude and affective experiences, in a lifespan sample.

### The relationship between solitude and affective experiences

Previous studies have differentiated solitude from social isolation and loneliness both theoretically and empirically (e.g., Campagne, 2019; Endo et al., 2017; Havens et al., 2004; Hoppmann et al., 2021). Solitude refers to the objective state of being alone (as we define it in the present study), which can be a positive, negative, or even conflicting experience, in the moments when it occurs. In contrast, social isolation and loneliness are typically associated exclusively with negative affect (Chappell & Badger, 1989; Wichers, 2014). Social isolation describes an objective scarcity of social networks and interactions (Cornwell & Waite, 2009) and is linked to stress, anxiety, and depression (Holmes et al., 2020). Loneliness, on the other hand, is defined as a subjective perception of lacking social contact (Perlman & Peplau, 1981). Similar to social isolation, loneliness is associated with depression, anxiety, and suicidal ideation (Beutel et al., 2017).

Previous studies tested associations between solitude and affective well‐being on the basis of the circumplex model of affect (Russell, 1980). Treating valence and arousal as two distinct dimensions, the model incorporates all affective states comprehensively within an orthogonal dimensional system. Solitude has been consistently found to be associated with a greater low‐arousal affect and less high‐arousal affect (e.g., Nguyen et al., 2018; Pauly et al., 2018). Specifically, individuals report lower levels of high‐arousal positive (HAP) states (e.g., excitement), higher levels of low‐arousal negative (LAN) states (e.g., sadness), and higher levels of low‐arousal positive (LAP) states (e.g., calm) after a 15‐minute solitude induction (Nguyen et al., 2018) and in moments of being alone (vs. with others) in daily life (Pauly et al., 2018). Findings on the relationship between solitude and high‐arousal negative (HAN) states (e.g., fear) are mixed. Nguyen et al. (2018) found a negative association between solitude and HAN in college student samples. Pauly et al. (2018) did not find a significant solitude‐HAN association in adult lifespan samples but found significant associations between solitude and higher levels of salivary cortisol and dehydroepiandrosterone sulfate (DHEAs), suggesting that individuals experience more stress in solitude compared to when with others. Solitude was also suggested to be associated with increased boredom (Wilson et al., 2014), anxiety (Rubin et al., 2010), and loneliness (Larson, 1990). To compare the current study findings with those of previous studies and to measure affective states comprehensively, we followed previous research (e.g., Nguyen et al., 2018; Pauly et al., 2018) by using the circumplex model of affect (Russell, 1980).

Previous research has investigated personal and interpersonal factors that moderate the association between solitude (defined either as being alone or the absence of social interaction) and affective experiences. In terms of personal factors, it has been found that individuals who were older (Jiang et al., 2024; Pauly et al., 2017), of East Asian heritage (versus European heritage; Jiang et al., 2019), or had higher levels of self‐efficacy or desire for solitude (Lay et al., 2019), reported more positive affective states in solitude. In terms of interpersonal factors, individuals who had more satisfied relationships (Choi et al., 2023; Pauly et al., 2018) or who suffered more interpersonal conflict (Choi et al., 2023) have been found to enjoy being alone more. Similarly, the positive association between solitude and negative experiences has been found to be weaker among individuals with more conflictual social relationships (Birditt et al., 2019) or higher levels of perceived social support (Fang et al., 2022). Previous research has shown that solitude creates a quiet space for self‐reflection, self‐evaluation, and concentration (Larson, 1990), which are related to the experience of state mindfulness (Soysal & Bakalım, 2024). However, one study found that individuals with higher trait self‐reflection experienced solitude negatively (Lay et al., 2019). Although solitude is conceptualized as a self‐reflective time that is theoretically relevant to the study of mindfulness (Cleveland, 2020; Soysal & Bakalım, 2024), to date, there is a lack of research examining trait mindfulness and its relation to affective experiences in solitude.

### The role of trait mindfulness

Mindfulness is defined as a conscious awareness of experiences (i.e., physical sensations, cognitions, emotions, and thoughts) in the present moment without judgment or reaction (Kabat‐Zinn, 1990). After examining the psychometric characteristics of available mindfulness questionnaires, Baer et al. (2006) found strong support for a five‐facet conceptualization of trait mindfulness. The five facets are *observing* (i.e., paying attention to internal and external experiences); *describing* (i.e., labeling or depicting experiences with words); *acting with awareness* (i.e., focusing entirely on one's current activity); *nonjudging of inner experiences* (i.e., being non‐evaluative toward thoughts and feelings); and *nonreactivity to inner experiences* (i.e., allowing thoughts and feelings to come and go without getting caught up in or carried away by them).

We expected trait mindfulness to moderate associations between being in solitude and affective experiences for two reasons. First, as social animals, solitude seems to go against human beings' innate need for social belonging and is therefore perceived and studied as a well‐being risk factor linked with loneliness and depression (Hawkley & Cacioppo, 2010; Long et al., 2003; Nguyen et al., 2019). Previous studies have shown that mindfulness plays a moderating role in the association between well‐being risk factors and cognitive and affective outcomes. For instance, a study targeting freshmen in university found that mindfulness practice lessened the association between loneliness and poor academic performance (Rosenstreich & Margalit, 2015). Lee and Zelman (2019) also found that a higher level of trait mindfulness alleviated the positive association between boredom proneness and negative affect (i.e., stress, anxiety, and depression). These findings suggest that mindfulness helps individuals pay attention to the present moment and decreases their worry about the past and future (Robins et al., 2004; Segal et al., 2004), which could be related to enhanced affective experiences in the circumstance of being alone. Mindfulness also allows individuals to rapidly become aware of current situations and to seek flexible coping strategies (Germer, 2005), thereby mitigating the negative influence of well‐being risk factors. Some studies have further investigated the moderating effects of specific facets of mindfulness on the association between risk factors and affective experiences. For example, Jin and Miao (2022) conducted a two‐wave longitudinal study and found that college students with higher levels of *acting with awareness* and *nonjudging of inner experiences* reported less negative affect during family incivility. However, a higher level of *observing* strengthened the positive association between family incivility and negative affect. Moreover, Lee and Zelman (2019) found that boredom proneness was positively associated with anxiety and stress, but only among individuals who were lower in the *acting with awareness* or *describing* facets of mindfulness. They also found boredom proneness was positively associated with depression, but only among individuals lower in *acting with awareness*. Based on these protective effects of trait mindfulness against negative affect, we expected that greater mindfulness would be associated with lower levels of negative affective experiences in solitude.

The second reason for our predicted moderating role of mindfulness is that, according to person‐environment fit theory, individuals enjoy better well‐being in situations that align with their traits (Edwards et al., 1998). The quietness of solitude can contribute to inner peace (Long et al., 2003) and self‐reflection (Bryant, 2013; Larson, 1990; Suedfeld et al., 1982), which are consistent with the nature of mindfulness. Moreover, it is possible that individuals high in trait mindfulness are better able to focus on the present moment and accept their current sensations and feelings openly and without evaluation, which helps them establish emotional balance and alleviate negative feelings. Cleveland (2020) also argued that solitude and mindfulness have three common characteristics: (1) both are autonomous choices made consciously by individuals instead of forced or compelled; (2) both represent the status of being‐with the self or the present; and (3) both contribute to self‐reconceptualization and better wellness. As a result, individuals with higher trait mindfulness may tend to experience more positive affect and less distress during time alone compared to those with lower trait mindfulness.

### The present study

Based on the established protective role of mindfulness and person‐environment fit theory, we investigated the moderating role of trait mindfulness on the solitude‐affect association. Specifically, we sought to understand how the five facets of mindfulness would moderate individuals' affective experiences in solitude. To maximize ecological validity, we adopted the experience sampling method; participants from an adult lifespan sample reported their momentary affect and social situations five times per day across a seven‐day period.

In line with previous research findings (Nguyen et al., 2018; Pauly et al., 2018), we hypothesized that individuals would report more and low‐arousal positive and negative affect (i.e., LAP and LAN affect) and less high‐arousal positive and negative affect (i.e., HAP and HAN affect) in moments of solitude compared to moments when with others (H1). Given the potential protective role of mindfulness, we expected the associations between solitude and LAP affect to be stronger, and the associations between solitude and HAP, HAN, and LAN affect to be weaker, in people with higher (vs. lower) levels of trait mindfulness (H2). We expected the moderating role of mindfulness to be consistent across the five facets (i.e., *observing*, *describing*, *acting with awareness*, *nonjudging of inner experiences*, and *nonreactivity to inner experiences*).

## METHOD

### Open Science statement

The study was not preregistered. However, data used is part of a study that was pre‐registered on the Open Science Framework (OSF) repository at https://osf.io/v4yca prior to data collection. Data and analysis code for the present study are provided on a separate Open Science Framework repository (https://osf.io/sn9h3/).

### Participants

A total of 188 participants (66.50% female, age range 19–93 years, *M*_*age*_ = 51.47 years, *SD*_*age*_ = 17.75 years) were recruited through various approaches, including sending the recruitment advertisement and posters to students and staff at a university and to community centers in Hong Kong. Among these participants, 13.30% were married, 21.81% had finished post‐secondary education, and 15.43% lived alone. A power analysis was conducted in R based on code from Kirtley et al. (2019). We planned a sampling design of 180 participants × 35 ESM assessments that would detect between‐person effect sizes of *β* = 0.10 with power = 0.90, *α*_2‐tailed_ = 0.05, by conducting 1,000 Monte Carlo simulations. To be eligible for the study, participants had to meet the following criteria: (a) born and raised in Hong Kong, (b) aged 18 years or above, (c) proficient or partially proficient skills in the use of mobile devices, with or without support, and (d) no major mental, physical, or cognitive problems (e.g., major depression, physical disability, stroke, etc.). All participants signed the informed consent forms and were debriefed after the study.

### Procedure

We used the experience sampling method (ESM) to collect participants' reports of their momentary social situations and affective states in real life. Participants first completed a questionnaire assessing demographic information and trait mindfulness. Participants were then asked to download PIEL (https://pielsurvey.org/), a smartphone‐based ESM application, and they learned how to use PIEL to complete the ESM questionnaires in a training session. During the seven‐day experience‐sampling phase, questionnaires were administered by PIEL at different times throughout the day in a pseudo‐random manner, within five 2‐hour blocks. Assessments were sent to participants at random times within each block, with a minimum of 1 hour between successive blocks. Participants who found PIEL difficult to use were allowed to respond to the questionnaires through the Qualtrics online survey platform instead. For participants who used Qualtrics to complete the ESM questionnaires, a research assistant first generated random times within each block and then sent links to the ESM study questionnaires to participants at these random times, five times per day, through the WhatsApp instant messenger app. In situations when participants did not respond to the initial notification from PIEL or WhatsApp, reminder notifications were sent after 10 minutes and after 20 minutes.

In the experience sampling questionnaires, participants were asked to report their momentary affective states, and then to report their social situations during the last 15 minutes. The assessment procedure yielded a maximum of 35 questionnaires for each participant. Responses that met the following criteria were included in the data analyses: (a) data on a particular day were included if the participant completed at least three questionnaires on that day; (b) data for a particular participant were included if the participant had completed at least 21 questionnaires in total (i.e., at least three responses each day). In the final sample, each participant's total number of ESM questionnaires ranged from 21 to 59, with a mean of 34.41. The numbers exceeding 35 occurred because some participants (*N* = 56) skipped one or two days in the first seven days, necessitating an extension of their ESM study period until they had finished the seven days. In total, 6,470 ESM questionnaires were included in the data analysis.

### Measures

#### Trait mindfulness

The Chinese version (Hou et al., 2014) of the Five Facet Mindfulness Questionnaire – short form (FFMQ‐SF; Baer et al., 2006) was used to measure trait mindfulness. The FFMQ‐SF contains 39 items measuring the five facets of trait mindfulness (i.e., *observing*, *describing*, *acting with awareness*, *nonjudging of inner experiences*, and *nonreactivity to inner experiences*). These items were scored on a 5‐point Likert scale (1 = *never or very rarely true*, 5 = *very often or always true*). The Omegas (as indices of reliability) for each of the FFMQ‐SF subscales are reported in Table 1.

**TABLE 1:** Descriptive results for central study variables and covariates.

Variables	Hong Kong Chinese participants (*N* = 188)		
	*Mean*	*SD*	*Omega (reliability)*
Level‐2 (person level)			
Age (19–93 years)	51.47	17.75	
Marital status (married %)	50.53		
Education (finished post‐secondary education %)	21.81		
Gender (female %)	66.50		
Living status (living alone %)	15		
Trait mindfulness facets			
Observing (1–5)	2.98	0.63	0.83
Describing (1–5)	3.12	0.47	0.66
Acting with awareness (1–5)	3.38	0.66	0.88
Nonjudging of inner experiences (1–5)	3.09	0.65	0.85
Nonreactivity to inner experiences (1–5)	3.05	0.52	0.75
Level‐1 (momentary level)			
Momentary solitude (solitude %)	26	44	
Momentary HAP affect (0–10)	5.44	2.43	
Momentary LAP affect (0–10)	6.18	2.24	
Momentary HAN affect (0–10)	2.20	2.06	
Momentary LAN affect (0–10)	3.57	2.15	

Abbreviations: HAN, high‐arousal negative; HAP, high‐arousal positive; LAN, low‐arousal negative; LAP, low‐arousal positive.

#### Momentary solitude

In each ESM study questionnaire, participants were asked to indicate whether they had interacted with anyone socially during the last 15 minutes (before they were beeped) by selecting one of three social situation options. Their social situation was coded as “solitude” when they selected “alone (no one nearby, no one you can see)”. The other two options ‐ “One or more people nearby who you were interacting with in‐person [talking or doing an activity together]” and “One or more people nearby who you were not interacting with in‐person” were coded as “non‐solitude”.

#### Momentary affect

Seven items from the momentary version of the affect valuation index (AVI; Tsai et al., 2006; Jiang et al., 2016) were used to measure momentary affective states. The seven items were “irritated,” “tired,” “relaxed,” “energized,” “anxious,” “calm,” and “bored.” Participants rated items on an 11‐point Likert scale (0 = *not at all*, 10 = *very much*). Based on the circumplex model of affect (Russell, 1980) and the AVI (Tsai et al., 2006), the affective states were categorized as high‐arousal positive affect (HAP; energized), low‐arousal positive affect (LAP; relaxed and calm; between‐person reliability estimate = 0.98; within‐person reliability estimate = 0.64), high‐arousal negative affect (HAN; irritated and anxious; between‐person reliability estimate = 0.89; within‐person reliability estimate = 0.56), and low‐arousal negative affect (LAN; tired and bored; between‐person reliability estimate = 0.66; within‐person reliability estimate = 0.33). Between‐ and within‐person reliabilities were calculated using HLM (Raudenbush et al., 2011) in Mplus (Muthén & Muthén, 1998–2021).

#### Demographic information

Age, gender (female = 1, male = 0), relationship status (married = 1, not married = 0), education level (finished post‐secondary education = 1, did not finish or attend post‐secondary education = 0), and living situation (living alone = 1, not living alone = 0) were measured in the demographics questionnaire.

### Data analysis

In the experience sampling data, sampling observations (Level 1, momentary level) were nested within participants (Level 2, person level). Hence, multilevel linear regression analyses were performed using the “lme4” package (Bates et al., 2015) in *R* to account for the multilevel nature of the data. The intercept was assumed to be random in the multilevel linear models, and a two‐level variance structure (person and momentary level) was adopted. Regression models were generated with HAP, LAP, HAN, and LAN as the outcome variables at Level 1. Momentary solitude (1 = solitude; 0 = non‐solitude) was the predictor at Level 1, and the five facets of trait mindfulness were the moderators at Level 2. The grand‐mean‐centered facets of mindfulness (i.e., *observing*, *describing*, *acting with awareness*, *nonjudging of inner experiences*, and *nonreactivity to inner experiences*) were entered into the models as moderators – each mindfulness facet was entered into a separate model, due to the potentially strong correlations among the mindfulness facets. Because age (e.g., Jiang et al., 2019; Lay et al., 2020; Pauly et al., 2017), education level (Jacobsen, 2004), marital status (Zhang & Li, 2021), and whether one is living alone (Simpson et al., 1993) are associated with affective experiences during solitude, they were included in the multilevel regression models as covariates at Level 2.

## RESULTS

Table 1 presents the sample demographic details, means, and standard deviations of IV, DVs, moderators, and covariates at Level 1 and Level 2. We first tested the relationship between being in solitude and affective experiences, and then tested whether these relationships were moderated by the five facets of mindfulness. The multilevel regression models are presented in Table 2. We report in Table 2 only those models for which the moderation effects were statistically significant; the remaining models are reported in the Supplementary Materials.

**TABLE 2:** Results of multilevel regression analyses.

Variable	Model 1: solitude as the only predictor Coefficient (SE)	Model 2: Main effects of mindfulness facets added Coefficient (SE)	Model 3: mindfulness interactions added Coefficient (SE)
*HAP affect: **Describing** as the moderator*			
Intercept	5.65*** (0.16)	5.67*** (0.15)	5.68*** (0.14)
Age	0.03*** (0.01)	0.03*** (0.01)	0.03*** (0.01)
Education level	−0.45 (0.25)	−0.48* (0.23)	−0.49* (0.23)
Marital status	−0.07 (0.35)	−0.01 (0.33)	−0.02 (0.33)
Living status	0.09 (0.45)	−0.19 (0.42)	−0.19 (0.42)
Solitude	−0.20*** (0.06)	−0.20*** (0.06)	−0.21*** (0.06)
Describing		1.17*** (0.22)	1.10*** (0.22)
Solitude × describing			0.25* (0.12)
ICC	0.40	0.36	0.36
Residual variance	3.27	3.27	3.26
Model fit (*R* ^*2*^ )	0.08	0.13	0.13
*HAP affect: **Acting with awareness** as the moderator*			
Intercept	5.65*** (0.16)	5.65*** (0.14)	5.65*** (0.14)
Age	0.03*** (0.01)	0.02** (0.01)	0.02** (0.01)
Education level	−0.45 (0.25)	−0.48* (0.23)	−0.48* (0.23)
Marital status	−0.07 (0.35)	0.14 (0.33)	0.13 (0.33)
Living status	0.09 (0.45)	−0.04 (0.42)	−0.04 (0.42)
Solitude	−0.20*** (0.06)	−0.20*** (0.06)	−0.21*** (0.06)
Acting with awareness		0.91*** (0.16)	0.86*** (0.16)
Solitude × acting with awareness			0.19* (0.09)
ICC	0.40	0.36	0.36
Residual variance	3.27	3.27	3.26
Model fit (*R* ^*2*^ )	0.08	0.13	0.13
*LAP affect: **Nonjudging of inner experiences** as the moderator*			
Intercept	6.40*** (0.16)	6.42*** (0.16)	6.42*** (0.16)
Age	0.01 (0.01)	0.00 (0.01)	0.00 (0.01)
Education level	−0.57* (0.27)	−0.57* (0.26)	−0.57* (0.26)
Marital status	−0.37 (0.37)	−0.49 (0.37)	−0.49 (0.37)
Living status	0.09 (0.48)	0.17 (0.47)	0.16 (0.47)
Solitude	0.17*** (0.05)	0.17*** (0.05)	0.16** (0.05)
Nonjudging of inner experiences		0.52** (0.18)	0.59** (0.19)
Solitude × nonjudging of inner experiences			−0.24** (0.08)
ICC	0.51	0.50	0.50
Residual variance	2.38	2.38	2.37
Model fit (*R* ^*2*^ )	0.03	0.06	0.06
*LAP affect: **Nonreactivity to inner experiences** as the moderator*			
Intercept	6.40*** (0.16)	6.40*** (0.16)	6.41*** (0.16)
Age	0.01 (0.01)	0.01 (0.01)	0.01 (0.01)
Education level	−0.57* (0.27)	−0.57* (0.26)	−0.58* (0.26)
Marital status	−0.37 (0.37)	−0.33 (0.37)	−0.34 (0.37)
Living status	0.09 (0.48)	−0.02 (0.48)	−0.04 (0.48)
Solitude	0.17*** (0.05)	0.17*** (0.05)	0.16** (0.05)
Nonreactivity to inner experiences		0.46* (0.23)	0.40 (0.23)
Solitude × nonreactivity to inner experiences			0.31** (0.11)
ICC	0.51	0.50	0.50
Residual variance	2.38	2.38	2.37
Model fit (*R* ^*2*^ )	0.03	0.05	0.05
*LAN affect: **Observing** as the moderator*			
Intercept	3.52*** (0.15)	3.52*** (0.15)	3.52*** (0.15)
Age	−0.02** (0.01)	−0.02**(0.01)	−0.02** (0.01)
Education level	0.29 (0.24)	0.29 (0.24)	0.29 (0.24)
Marital status	−0.42 (0.34)	−0.42 (0.34)	−0.43 (0.34)
Living status	−0.30 (0.44)	−0.30 (0.44)	−0.30 (0.44)
Solitude	−0.00 (0.05)	−0.00 (0.05)	0.00 (0.05)
Observing		0.02 (0.17)	0.08 (0.17)
Solitude × observing			−0.24** (0.08)
ICC	0.47	0.47	0.46
Residual variance	2.33	2.33	2.32
Model fit (*R* ^*2*^ )	0.05	0.05	0.05
*LAN affect: **Nonreactivity to inner experiences** as the moderator*			
Intercept	3.52*** (0.15)	3.52*** (0.15)	3.52*** (0.15)
Age	−0.02** (0.01)	−0.02** (0.01)	−0.02** (0.01)
Education level	0.29 (0.24)	0.29 (0.24)	0.29 (0.24)
Marital status	−0.42 (0.34)	−0.43 (0.34)	−0.42 (0.34)
Living status	−0.30 (0.44)	−0.28 (0.44)	−0.26 (0.44)
Solitude	−0.00 (0.05)	−0.00 (0.05)	0.01 (0.05)
Nonreactivity to inner experiences		−0.08 (0.21)	−0.03 (0.21)
Solitude × nonreactivity to inner experiences			−0.27** (0.10)
ICC	0.47	0.47	0.46
Residual variance	2.33	2.33	2.32
Model fit (*R* ^*2*^ )	0.05	0.05	0.05

*Note*: N = 188.

Abbreviations: HAP, high‐arousal positive; ICC, intraclass correlation; LAN, low‐arousal negative; LAP, low‐arousal positive; SE, Standard error.

* *p* < 0.05.

** *p* < 0.01.

*** *p* < 0.001.

### Affective experiences in solitude

Results showed that participants reported significantly less HAP (*b* = −0.21, *SE* = 0.06, *t* = −3.50, *p* < 0.001) and more LAP (*b* = 0.17, *SE* = 0.05, *t* = 3.26, *p* = 0.001) when they were alone (vs. with others) in the last 15 minutes. There was no significant relationship between being in solitude and HAN or LAN affect.

### The moderating role of mindfulness on affective experiences in solitude

Consistent with H2, we found that four of the five facets of mindfulness (i.e., observing, describing, acting with awareness, and nonreactivity to inner experiences) were associated with better affective experiences in solitude. The specific results are presented below according to the four categories of affective states.

#### HAP affect

**FIGURE 1:**
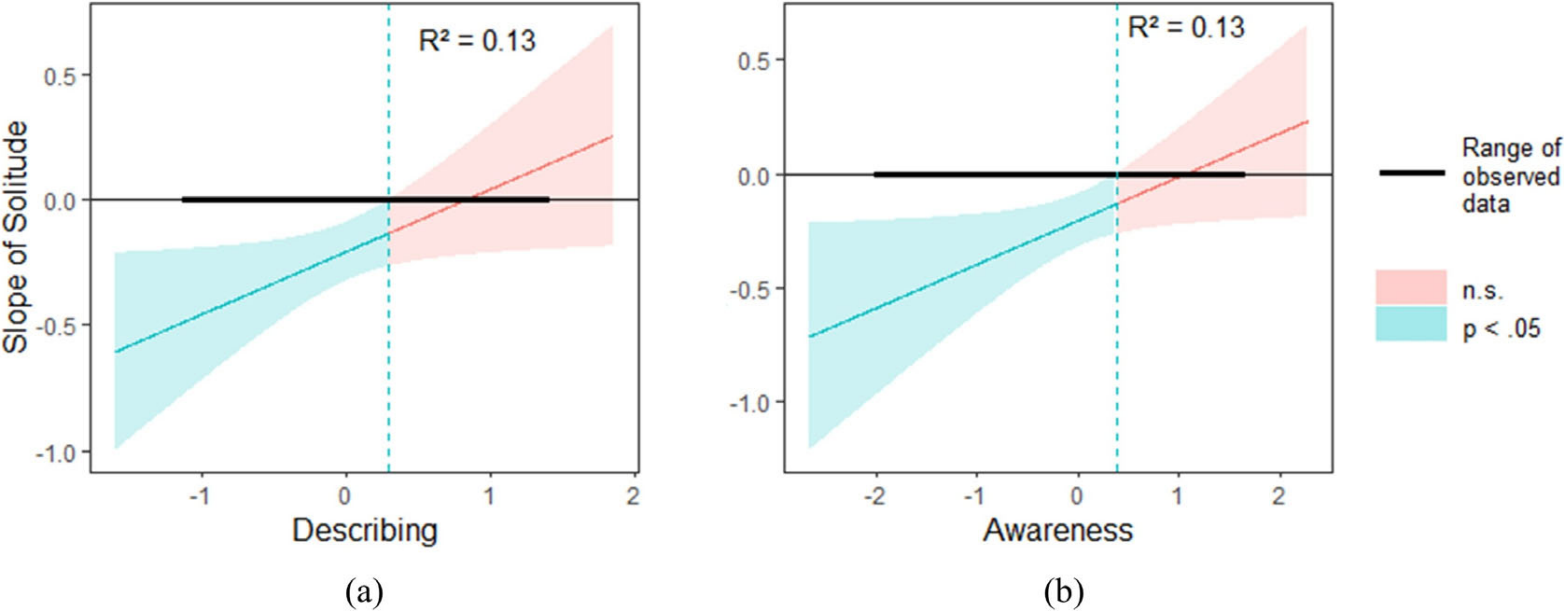
The Johnson‐Neyman plot showing the interaction between solitude and describing (a), as well as the interaction between solitude and acting with awareness (b), when HAP affect was entered as the dependent variable.

*Describing* moderated the association between solitude and HAP affect (*b* = 0.25, *SE* = 0.12, *t* = 2.11, *p* = 0.035). Simple slope analysis showed that being in solitude was associated with less HAP affect for people who reported a lower level of *describing* (Mean ‐1SD; *b* = −0.33, *SE* = 0.08, *t* = −3.87, *p* < 0.001), whereas this association was not significant for those who reported a higher level of *describing*, (Mean + 1SD; *b* = −0.09, *SE* = 0.08, *t* = −1.10, *p* = 0.271). We used the “*interactions*” package in R to plot a Johnson – Neyman Plot to show the region of significance. The blue vertical dashed lines represent the separation between significant and nonsignificant regions (Figure 1a).

*Acting with awareness* moderated the relationship between solitude and HAP affect (*b* = 0.19, *SE* = 0.09, *t* = 2.09, *p* = 0.036). Simple slope analysis showed that being in solitude was associated with less HAP affect for people who reported a lower level of *acting with awareness* (*b* = −0.33, *SE* = 0.09, *t* = −3.81, *p* < 0.001), whereas this association was not significant for those who reported a higher level of *acting with awareness* (*b* = −0.08, *SE* = 0.08, *t* = −0.96, *p* = 0.337; Figure 1b).

#### LAP affect

**FIGURE 2:**
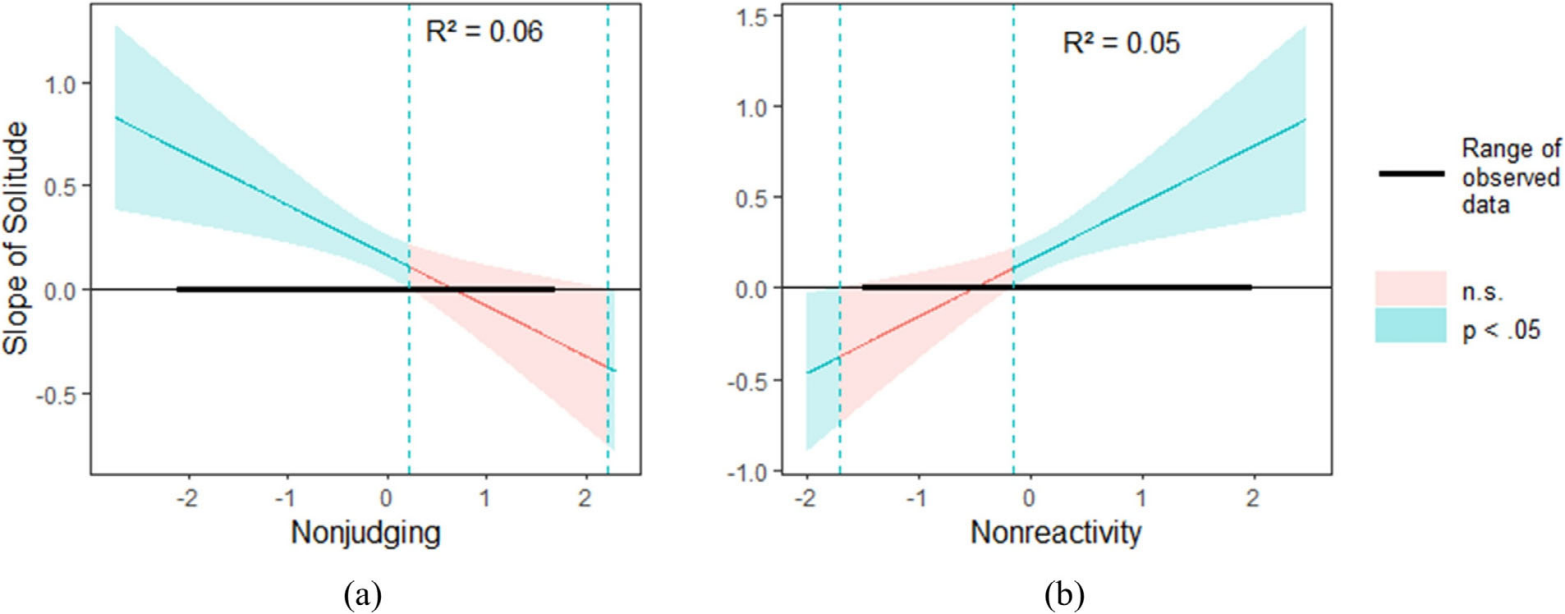
The Johnson‐Neyman plot showing the interaction between solitude and nonjudging of inner experiences (a), as well as the interaction between solitude and nonreactivity to inner experiences (b), when LAP affect was entered as the dependent variable.

*Nonjudging of inner experiences* moderated the association between solitude and LAP affect (*b* = −0.24, *SE* = 0.08, *t* = −2.95, *p* = 0.003). Simple slope analysis showed that being in solitude was associated with more LAP for people who reported a lower level of *nonjudging of inner experiences* (*b* = 0.32, *SE* = 0.07, *t* = 4.44, *p* < 0.001), whereas this association was not significant for those who reported a higher level of *nonjudging of inner experiences* (*b* = 0.01, *SE* = 0.08, *t* = 0.07, *p* = 0.947) (Figure 2a).

*Nonreactivity to inner experiences* moderated the relationship between solitude and LAP affect (*b* = 0.31, *SE* = 0.11, *t* = 2.95, *p* = 0.003). Simple slope analysis showed that being in solitude was associated with more LAP affect for people who reported a higher level of *nonreactivity to inner experiences* (*b* = 0.32, *SE* = 0.07, *t* = 4.43, *p* < 0.001), whereas this association was not significant for those who reported a lower level of *nonreactivity to inner experiences* (*b* = −0.01, *SE* = 0.08, *t* = −0.07, *p* = 0.941; Figure 2b).

#### HAN affect

None of the five facets of mindfulness moderated the association between solitude and HAN affect.

#### LAN affect

**FIGURE 3:**
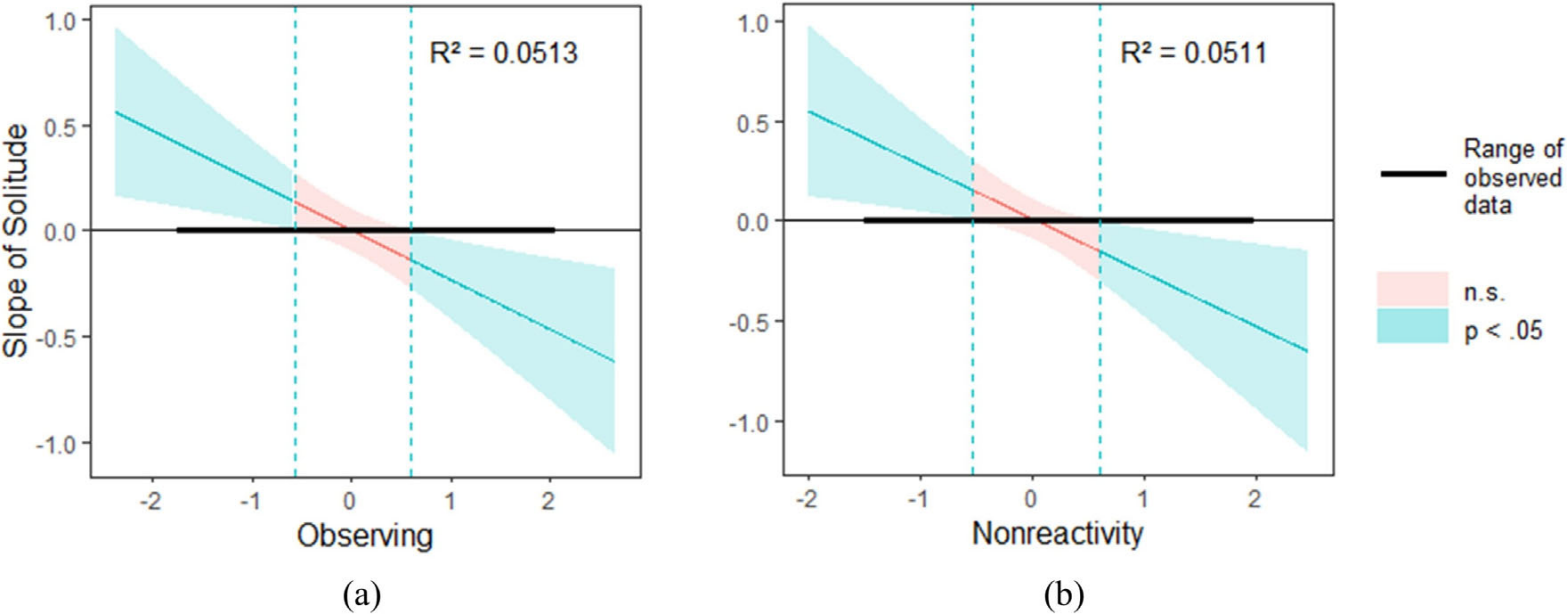
The Johnson‐Neyman plot showing the interaction between solitude and observing (a), as well as the interaction between solitude and nonreactivity to inner experiences (b), when LAN affect was entered as the dependent variable.

*Observing* moderated the relationship between solitude and LAN affect (*b* = −0.24, *SE* = 0.08, *t* = −2.84, *p* = 0.005). Simple slope analysis showed that being in solitude was associated with more LAN affect for people who reported a lower level of *observing* (*b* = 0.16, *SE* = 0.07, *t* = 2.10, *p* = 0.036), and was associated with less LAN affect for people who reported a higher level of *observing* (*b* = −0.15, *SE* = 0.07, *t* = −2.09, *p* = 0.037; Figure 3a).

*Nonreactivity to inner experiences* moderated the association between solitude and LAN affect (*b* = −0.27, *SE* = 0.10, *t* = −2.58, *p* = 0.010). Simple slope analysis showed that being in solitude was associated with more LAN affect for people who reported a lower level of *nonreactivity to inner experiences* (*b* = 0.15, *SE* = 0.08, *t* = 1.96, *p* = 0.050), whereas this association was not significant for those who reported a higher level of *nonreactivity to inner experiences* (*b* = −0.13, *SE* = 0.07, *t* = −1.87, *p* = 0.062; Figure 3b).

Given the two‐way Solitude X mindfulness facet interactions with HAN affect as the DV were all nonsignificant, we reported them in the Supplementary Materials (see Table S1).

## DISCUSSION

In this experience‐sampling study, we investigated the association between solitude and affective experiences and the moderating role of five facets of trait mindfulness in an adult lifespan sample. We found that higher trait mindfulness was linked with a strengthened positive association between solitude and affective well‐being. Specifically, higher levels of *describing* and *acting with awareness* were linked with a weakened negative association between being in solitude and HAP affect. Moreover, a higher level of *nonreactivity to inner experiences* was linked with a strengthened positive association between solitude and LAP affect and a weakened positive association between solitude and LAN affect. A higher level of *observing* was also linked with a weakened positive association between solitude and LAN affect. The only unexpected negative effect of mindfulness was that a higher level of *nonjudging of inner experiences* was linked with a weakened positive association between solitude and LAP affect. The above results suggest that trait mindfulness shows a moderating effect on affective experiences during periods of solitude versus being with others through its various facets.

### Solitude is associated with low‐arousal affective states

Our findings revealed that spending time alone was linked with reduced HAP and increased LAP, which is partly consistent with the hypotheses. This result suggests that people report feeling peaceful, rather than excited, in solitude. Previous research has reached a parallel conclusion, suggesting that being in solitude is accompanied by low‐arousal positive affect (Lay et al., 2019). Solitude showed no direct effects on high and low‐arousal negative affect in this study, which is inconsistent with previous studies (e.g., Nguyen et al., 2018; Rodriguez et al., 2025). Many studies suggest that solitude is associated with greater LAN (Matias et al., 2011; Pauly et al., 2017; Pauly et al., 2018), whereas its association with HAN has been mixed. Whether solitude is perceived as positive or negative depends in part on activities during solitude (Long et al., 2003). The present study did not distinguish the specific activities individuals engaged in when they were alone. It is possible that, because individuals engage in a variety of activities and have a variety of experiences in solitude (such as solitary meditation, artistic creation, and being alone due to social exclusion), this may lead to mixed impacts of solitude on both LAN and HAN affect. Future studies may consider specifying activities engaged in during solitude to specify the effect of solitude on HAN and LAN affect.

### Higher trait mindfulness strengthens positive associations between solitude and positive affect

In general, higher levels (vs. lower levels) of trait mindfulness were linked with amplified positive associations between solitude and positive affect, and diminished positive associations between solitude and negative affect. The five facets of mindfulness showed distinct relationships with different categories of affective states. People with lower levels of *observing* reported increased LAN affect, whereas those with higher levels of *observing* reported decreased LAN affect in solitude (vs. non‐solitude), suggesting that sufficient skills in *observing* could assist in shifting one's mindset to have more positive experiences of solitude. These results validate previous findings suggesting that *observing* is correlated with increased personal growth and autonomy by assisting individuals to acquire a deeper and more positive understanding of life (Iani et al., 2017). *Observing* actively directs people's attention to their physical sensations and perceptions of the external world in moments of solitude, which can assist them in fully experiencing their feelings of distress in the moment and then managing these feelings, resulting in lowered negative affect (Dimidjian & Linehan, 2003; Roemer et al., 2021).

The differences in HAP affect between solitude and non‐solitude situations were smaller for individuals with higher levels of *describing* and *acting with awareness*, compared to those with lower levels of the two mindfulness facets. This finding reveals that mindfulness is associated with more positive affect (specifically, higher levels of energy) during periods of being alone. It is consistent with previous studies that found *describing* accounted for improved well‐being and *acting with awareness* was a strong negative predictor of distress (Roemer et al., 2021).

Unexpectedly, a weakened positive association between solitude and LAP was found among people with higher levels of *nonjudging of inner experiences* rather than those with lower levels of *nonjudging*. This result failed to support our hypothesis and previous research (e.g., Cash & Whittingham, 2010; Ortet et al., 2020). However, this finding might be explained by the speculation that individuals who struggle to maintain a nonjudging perspective may utilize various emotion regulation strategies (e.g., cognitive reappraisal, expressive suppression, etc.) to regulate their negative affect in solitude. The appropriate use of emotion regulation strategies could improve mental state and enhance happiness (Chen et al., 2022; Extremera & Rey, 2015). On the other hand, being skilled in *nonjudging of inner experiences* facilitates the process of attending to the present moment but may be associated with passive coping (Brown et al., 2007). Hence, this could explain why a lower level of *nonjudging of inner experiences* is associated with greater low‐arousal positive affect in solitude.

*Nonreactivity to inner experiences* showed a moderating effect on both LAP and LAN affect in solitude. Individuals with higher *nonreactivity* reported increased LAP affect in solitude (vs. non‐solitude), while those with lower *nonreactivity* reported increased LAN affect in solitude (vs. non‐solitude). These results revealed that *nonreactivity* is linked with more positive solitude experiences, which aligns with the finding that *nonreactivity* is associated with more happiness (Roemer et al., 2021). Individuals with higher *nonreactivity to inner experiences* have an attitude of acceptance toward their physical sensations, thoughts, and affective experiences (Bishop et al., 2004). Not reacting to internal experiences allows individuals to calm down and experience more LAP affective states. Simultaneously, nonreactive awareness is associated with better adoption of adaptive psychological coping resources, which, in turn, is associated with more positive and less negative affect (Carmody et al., 2008; Roemer et al., 2021).

### Theoretical implications

The current study findings have important theoretical implications for research focusing on solitude, mindfulness, and affective experiences. Our study investigated the moderating roles of different facets of mindfulness on affective experiences in solitude using a lifespan sample, based on the shared attributes of mindfulness and solitude. To our knowledge, a very limited number of studies have examined affective experiences in solitude in the context of trait mindfulness. Our results make important theoretical contributions to our knowledge of the factors that influence affective experiences in solitude by highlighting the significance of trait mindfulness. The overall pattern of results suggests that trait mindfulness plays a vital role in soothing negative feelings and amplifying positive affect when people are physically alone. In addition, our study revealed that different facets of mindfulness play distinct roles. Specifically, *observing*, *describing*, *acting with awareness*, and *nonreactivity to inner experiences* exhibited positive effects on affective experiences in solitude (either by being associated with improved positive affect or by being associated with reduced negative affect), while *nonjudging of inner experiences* attenuated the positive link between solitude and positive affect. These findings suggest that the distinct roles of different facets of mindfulness should be examined in future studies.

### Limitations and future directions

Several limitations of the present study need to be addressed in future studies. Firstly, we defined solitude in the current study as being physically alone, without testing whether participants were interacting with others virtually (Nguyen & Rodriguez, 2024). While this definition has been widely adopted in previous research (e.g., Nguyen et al., 2018; Pauly et al., 2017; Pauly et al., 2018; Rodriguez et al., 2025), some studies have employed alternative definitions highlighting the subjective characteristics of solitude. For instance, subjective solitude has been described as “disengaging from social demands and surveillance”, which can be experienced in the presence of others (Larson, 1990; Long & Averill, 2003). Coplan et al. (2022) proposed the concept of “solitude gradient”, arguing that the level of solitude varies because of different degrees of digital technologies engagement, which is very common in modern life. It is possible that the associations between solitude and affective states may vary depending on the definition used. However, to the best of our knowledge, no studies have directly addressed this question. Future research should compare and contrast the relationships between solitude and affective states across different conceptualizations of solitude. This will help validate our findings on the moderating role of mindfulness in this relationship. Secondly, although this study's measures of affect have been validated (Russell, 1980; Tsai et al., 2006) and widely used in previous studies (e.g., Lay et al., 2024; Tsai et al., 2018), the within‐person reliabilities of the HAN affect and LAN affect measures in our sample are relatively low. Future studies may try to improve the within‐person reliability by extending the ESM period. Thirdly, our study investigated the impact of trait mindfulness on daily affective experiences in solitude contexts. Previous studies have found that state and trait mindfulness are two distinct constructs (Medvedev et al., 2017), hence, further exploration of the moderating role of state mindfulness on affective experiences would help develop a comprehensive understanding of the role of mindfulness in solitude experiences. Fourthly, previous studies have shown that affective experiences in solitude differ by culture and ethnicity (e.g., Jiang et al., 2015; Jiang et al., 2019; Jiang & Fung, 2019; Lay et al., 2020). It has been shown that individuals from individualistic (vs. collectivistic) cultures have a stronger preference for solitude in these works. Some studies have indicated that there is also cultural diversity in the role of mindfulness in daily life (e.g., Chen & Murphy, 2019; Sumell et al., 2021). However, research exploring the moderating role of mindfulness on solitude‐affect associations across cultures is limited. Only one study, targeting Chinese adolescents, showed that lower levels of mindfulness were associated with stronger positive associations between solitude and psychological distress (Lian et al., 2023), which aligns with our present findings. The role of mindfulness in the association between solitude and affective experiences may differ across cultural backgrounds or ethnicities (Fung & Jiang, 2016), which could be further examined in future work. Fifthly, the present study applied a micro‐longitudinal design to investigate naturally occurring correlational relationships between mindfulness, solitude, and affective experiences. Future experimental studies should examine causal links between these variables, for example, using mindfulness interventions or manipulation of social contexts. Finally, sample selectivity may introduce biases in the results. Samples in this study are possibly relatively tech‐savvy and healthy. While we have made efforts to use diverse recruitment strategies to ensure our sample is as representative as possible, the findings should be further validated in additional samples.

### Conclusion

The present study investigated affective experiences in moments of solitude compared to moments of being with others, and the moderating effects of trait mindfulness facets, using the experience sampling method with an adult lifespan sample. This sample reported significantly lower HAP and greater LAP in solitude (vs. non‐solitude). It was also found that trait mindfulness facets moderated some of the associations between solitude and affective experiences. Specifically, higher levels of *describing* and *acting with awareness* buffered against the increases in HAP affect in solitude. A higher level of *nonreactivity to inner experiences* was also linked with improved affective experiences in solitude through increased LAP affect and the absence of increased LAN affect in solitude. A higher level of *observing* was also associated with improved affective experiences through the reduction of LAN affect in solitude. Unexpectedly, a higher level of *nonjudging of inner experiences* was associated with worsened affective experiences, as it prevented increases in LAP affect in solitude. Our findings highlight the roles of different facets of mindfulness in the relationship between being in solitude and affective experiences across adulthood.

## CONFLICT OF INTEREST STATEMENT

The authors declare that they have no competing interests.

## ETHICS STATEMENT

All provided written informed consent, and the study procedures were approved by the Human Research Ethics Committee of the Education University of Hong Kong (Ref Number: 2020‐2021‐0165).

## Supporting information


**Table S1.** *Results of multilevel regression models with insignificant two‐way interactions*.

## Data Availability

The study was not preregistered. However, data used is part of a study that was pre‐registered on the Open Science Framework (OSF) repository at https://osf.io/v4yca prior to data collection. Data and analysis code for the present study are provided on a separate Open Science Framework repository (https://osf.io/sn9h3/).
