# Supplementary material for: Momentary solitude and affective experiences: The moderating role of trait mindfulness
Source: Appl Psychol Health Well Being. 2025 Jun 12;17(3):e70052. doi: 10.1111/aphw.70052 (PMC12160072; doi:10.1111/aphw.70052)
Supplement: Supplementary file 1 — Table S1. Results of multilevel regression models with insignificant two‐way interactions. [file APHW-17-0-s001.docx]

**Table S1**

*Results of multilevel regression models with insignificant two-way interactions*

| Variable | Model 1: Solitude as the only predictor  Coefficient (SE) | Model 2: Main effects of mindfulness facets added  Coefficient (SE) | Model 3: Mindfulness interactions added  Coefficient (SE) |
| --- | --- | --- | --- |
| *HAP affect:* ***observing*** *as the moderator* |  |  |  |
| Intercept | 5.65^***^ (0.16) | 5.67^***^ (0.15) | 5.67^***^ (0.15) |
| Age | 0.03^***^ (0.01) | 0.03^***^ (0.01) | 0.03^***^ (0.01) |
| Education level | -0.45 (0.25) | -0.45 (0.25) | -0.45 (0.25) |
| Marital status | -0.07 (0.35) | -0.08 (0.34) | -0.08 (0.34) |
| Living status | 0.09 (0.45) | -0.10 (0.45) | -0.09 (0.45) |
| Solitude | -0.20^***^ (0.06) | -0.20^***^ (0.06) | -0.20^***^ (0.06) |
| Observing |  | 0.48^**^ (0.17) | 0.46^**^ (0.17) |
| Solitude × observing |  |  | 0.06 (0.10) |
| ICC | 0.40 | 0.39 | 0.39 |
| Residual variance | 3.27 | 3.27 | 3.27 |
| Model fit (*R^2^*) | 0.08 | 0.09 | 0.09 |
| *HAP affect:* ***nonjudging of inner experiences*** *as the moderator* |  |  |  |
| Intercept | 5.65^***^ (0.16) | 5.66^***^ (0.15) | 5.66^***^ (0.15) |
| Age | 0.03^***^ (0.01) | 0.03^***^ (0.01) | 0.03^***^ (0.01) |
| Education level | -0.45 (0.25) | -0.44 (0.25) | -0.44 (0.25) |
| Marital status | -0.07 (0.35) | -0.14 (0.35) | -0.14 (0.35) |
| Living status | 0.09 (0.45) | 0.14 (0.45) | 0.14 (0.45) |
| Solitude | -0.20^***^ (0.06) | -0.20^***^ (0.06) | -0.20^***^ (0.06) |
| Nonjudging of inner experiences |  | 0.31 (0.18) | 0.31 (0.18) |
| Solitude × nonjudging of inner experiences |  |  | -0.01 (0.10) |
| ICC | 0.40 | 0.39 | 0.39 |
| Residual variance | 3.27 | 3.27 | 3.27 |
| Model fit (*R^2^*) | 0.08 | 0.09 | 0.09 |
| *HAP affect:* ***nonreactivity to inner experiences*** *as the moderator* |  |  |  |
| Intercept | 5.65^***^ (0.16) | 5.65^***^ (0.15) | 5.65^***^ (0.15) |
| Age | 0.03^***^ (0.01) | 0.03^***^ (0.01) | 0.03^***^ (0.01) |
| Education level | -0.45 (0.25) | -0.45 (0.24) | -0.45 (0.24) |
| Marital status | -0.07 (0.35) | -0.01 (0.34) | -0.02 (0.34) |
| Living status | 0.09 (0.45) | -0.08 (0.44) | -0.09 (0.44) |
| Solitude | -0.20^***^ (0.06) | -0.20^***^ (0.06) | -0.21^***^ (0.06) |
| Nonreactivity to inner experiences |  | 0.68^**^ (0.21) | 0.66^**^ (0.21) |
| Solitude × nonreactivity to inner experiences |  |  | 0.12 (0.12) |
| ICC | 0.40 | 0.38 | 0.38 |
| Residual variance | 3.27 | 3.27 | 3.27 |
| Model fit (*R^2^*) | 0.08 | 0.10 | 0.10 |
| *LAP affect:* ***observing*** *as the moderator* |  |  |  |
| Intercept | 6.40^***^ (0.16) | 6.42^***^ (0.16) | 6.42^***^ (0.16) |
| Age | 0.01 (0.01) | 0.01 (0.01) | 0.01 (0.01) |
| Education level | -0.57^*^ (0.27) | -0.58^*^ (0.26) | -0.58^*^ (0.26) |
| Marital status | -0.37 (0.37) | -0.38 (0.36) | -0.38 (0.36) |
| Living status | 0.09 (0.48) | -0.10 (0.47) | -0.11 (0.47) |
| Solitude | 0.17^***^ (0.05) | 0.17^***^ (0.05) | 0.17^***^ (0.05) |
| Observing |  | 0.51^**^ (0.18) | 0.49^**^ (0.18) |
| Solitude × observing |  |  | 0.11 (0.08) |
| ICC | 0.51 | 0.50 | 0.50 |
| Residual variance | 2.38 | 2.38 | 2.38 |
| Model fit (*R^2^*) | 0.03 | 0.05 | 0.05 |
| *LAP affect:* ***describing*** *as the moderator* |  |  |  |
| Intercept | 6.40^***^ (0.16) | 6.42^***^ (0.15) | 6.42^***^ (0.15) |
| Age | 0.01 (0.01) | 0.01 (0.01) | 0.01 (0.01) |
| Education level | -0.57^*^ (0.27) | -0.61^*^ (0.25) | -0.61^*^ (0.25) |
| Marital status | -0.37 (0.37) | -0.31 (0.35) | -0.31 (0.35) |
| Living status | 0.09 (0.48) | -0.20 (0.45) | -0.20 (0.45) |
| Solitude | 0.17^***^ (0.05) | 0.17^***^ (0.05) | 0.17^***^ (0.05) |
| Describing |  | 1.21^***^ (0.23) | 1.19^***^ (0.23) |
| Solitude × describing |  |  | 0.05 (0.10) |
| ICC | 0.51 | 0.47 | 0.47 |
| Residual variance | 2.38 | 2.38 | 2.38 |
| Model fit (*R^2^*) | 0.03 | 0.10 | 0.10 |
| *LAP affect:* ***acting with awareness*** *as the moderator* |  |  |  |
| Intercept | 6.40^***^ (0.16) | 6.39^***^ (0.15) | 6.39^***^ (0.15) |
| Age | 0.01 (0.01) | -0.00 (0.01) | -0.00 (0.01) |
| Education level | -0.57^*^ (0.27) | -0.62^**^ (0.24) | -0.62^**^ (0.24) |
| Marital status | -0.37 (0.37) | -0.11 (0.33) | -0.11 (0.33) |
| Living status | 0.09 (0.48) | -0.08 (0.43) | -0.08 (0.43) |
| Solitude | 0.17^***^ (0.05) | 0.17^***^ (0.05) | 0.17^***^ (0.05) |
| Acting with awareness |  | 1.17^***^ (0.17) | 1.16^***^ (0.17) |
| Solitude × acting with awareness |  |  | 0.05 (0.08) |
| ICC | 0.51 | 0.45 | 0.45 |
| Residual variance | 2.38 | 2.38 | 2.38 |
| Model fit (*R^2^*) | 0.03 | 0.14 | 0.14 |
| *HAN affect:* ***observing*** *as the moderator* |  |  |  |
| Intercept | 2.23^***^ (0.16) | 2.23^***^ (0.16) | 2.23^***^ (0.16) |
| Age | -0.01 (0.01) | -0.01 (0.01) | -0.01 (0.01) |
| Education level | 0.15 (0.26) | 0.15 (0.26) | 0.15 (0.26) |
| Marital status | -0.53 (0.36) | -0.53 (0.36) | -0.53 (0.36) |
| Living status | -0.39 (0.46) | -0.42 (0.47) | -0.42 (0.47) |
| Solitude | -0.07 (0.05) | -0.07 (0.05) | -0.07 (0.05) |
| Observing |  | 0.06 (0.18) | 0.07 (0.18) |
| Solitude × observing |  |  | -0.00 (0.07) |
| ICC | 0.56 | 0.56 | 0.56 |
| Residual variance | 1.82 | 1.82 | 1.82 |
| Model fit (*R^2^*) | 0.02 | 0.02 | 0.02 |
| *HAN affect:* ***describing*** *as the moderator* |  |  |  |
| Intercept | 2.23^***^ (0.16) | 2.22^***^ (0.16) | 2.22^***^ (0.16) |
| Age | -0.01 (0.01) | -0.01 (0.01) | -0.01 (0.01) |
| Education level | 0.15 (0.26) | 0.16 (0.25) | 0.16 (0.25) |
| Marital status | -0.53 (0.36) | -0.55 (0.36) | -0.56 (0.36) |
| Living status | -0.39 (0.46) | -0.29 (0.46) | -0.29 (0.46) |
| Solitude | -0.07 (0.05) | -0.07 (0.05) | -0.08 (0.05) |
| Describing |  | -0.43 (0.24) | -0.43 (0.24) |
| Solitude × describing |  |  | 0.08 (0.09) |
| ICC | 0.56 | 0.55 | 0.55 |
| Residual variance | 1.82 | 1.82 | 1.82 |
| Model fit (*R^2^*) | 0.02 | 0.03 | 0.03 |
| *HAN affect:* ***acting with awareness*** *as the moderator* |  |  |  |
| Intercept | 2.23^***^ (0.16) | 2.24^***^ (0.15) | 2.24^***^ (0.15) |
| Age | -0.01 (0.01) | -0.00 (0.01) | -0.00 (0.01) |
| Education level | 0.15 (0.26) | 0.19 (0.23) | 0.19 (0.23) |
| Marital status | -0.53 (0.36) | -0.76^*^ (0.33) | -0.76^*^ (0.33) |
| Living status | -0.39 (0.46) | -0.24 (0.42) | -0.24 (0.42) |
| Solitude | -0.07 (0.05) | -0.07 (0.05) | -0.07 (0.05) |
| Acting with awareness |  | -1.01^***^ (0.16) | -1.01^***^ (0.16) |
| Solitude × acting with awareness |  |  | -0.01 (0.07) |
| ICC | 0.56 | 0.51 | 0.51 |
| Residual variance | 1.82 | 1.82 | 1.82 |
| Model fit (*R^2^*) | 0.02 | 0.12 | 0.12 |
| *HAN affect:* ***nonjudging of inner experiences*** *as the moderator* |  |  |  |
| Intercept | 2.23^***^ (0.16) | 2.20^***^ (0.15) | 2.20^***^ (0.15) |
| Age | -0.01 (0.01) | -0.01 (0.01) | -0.01 (0.01) |
| Education level | 0.15 (0.26) | 0.14 (0.24) | 0.14 (0.24) |
| Marital status | -0.53 (0.36) | -0.34 (0.34) | -0.34 (0.34) |
| Living status | -0.39 (0.46) | -0.52 (0.44) | -0.52 (0.44) |
| Solitude | -0.07 (0.05) | -0.07 (0.05) | -0.07 (0.05) |
| Nonjudging of inner experiences |  | -0.84^***^ (0.17) | -0.84^***^ (0.17) |
| Solitude × nonjudging of inner experiences |  |  | 0.00 (0.07) |
| ICC | 0.56 | 0.53 | 0.53 |
| Residual variance | 1.82 | 1.82 | 1.82 |
| Model fit (*R^2^*) | 0.02 | 0.09 | 0.09 |
| *HAN affect:* ***nonreactivity to inner experiences*** *as the moderator* |  |  |  |
| Intercept | 2.23^***^ (0.16) | 2.23^***^ (0.16) | 2.23^***^ (0.16) |
| Age | -0.01 (0.01) | -0.02 (0.01) | -0.02 (0.01) |
| Education level | 0.15 (0.26) | 0.15 (0.26) | 0.15 (0.26) |
| Marital status | -0.53 (0.36) | -0.54 (0.36) | -0.54 (0.36) |
| Living status | -0.39 (0.46) | -0.37 (0.47) | -0.37 (0.47) |
| Solitude | -0.07 (0.05) | -0.07 (0.05) | -0.07 (0.05) |
| Nonreactivity to inner experiences |  | -0.08 (0.22) | -0.08 (0.22) |
| Solitude × nonreactivity to inner experiences |  |  | 0.01 (0.09) |
| ICC | 0.56 | 0.56 | 0.56 |
| Residual variance | 1.82 | 1.82 | 1.82 |
| Model fit (*R^2^*) | 0.02 | 0.02 | 0.02 |
| *LAN affect:* ***describing*** *as the moderator* |  |  |  |
| Intercept | 3.52^***^ (0.15) | 3.51^***^ (0.15) | 3.51^***^ (0.15) |
| Age | -0.02^**^ (0.01) | -0.02^**^ (0.01) | -0.02^**^ (0.01) |
| Education level | 0.29 (0.24) | 0.30 (0.24) | 0.31 (0.24) |
| Marital status | -0.42 (0.34) | -0.45 (0.33) | -0.45 (0.33) |
| Living status | -0.30 (0.44) | -0.15 (0.43) | -0.15 (0.43) |
| Solitude | -0.00 (0.05) | -0.00 (0.05) | 0.00 (0.05) |
| Describing |  | -0.60^**^ (0.22) | -0.56^*^ (0.22) |
| Solitude × describing |  |  | -0.15 (0.10) |
| ICC | 0.47 | 0.46 | 0.46 |
| Residual variance | 2.33 | 2.33 | 2.33 |
| Model fit (*R^2^*) | 0.05 | 0.07 | 0.07 |
| *LAN affect:* ***acting with awareness*** *as the moderator* |  |  |  |
| Intercept | 3.52^***^ (0.15) | 3.53^***^ (0.14) | 3.53^***^ (0.14) |
| Age | -0.02^**^ (0.01) | -0.02^*^ (0.01) | -0.02^*^ (0.01) |
| Education level | 0.29 (0.24) | 0.32 (0.23) | 0.32 (0.23) |
| Marital status | -0.42 (0.34) | -0.62 (0.32) | -0.61 (0.32) |
| Living status | -0.30 (0.44) | -0.17 (0.41) | -0.17 (0.41) |
| Solitude | -0.00 (0.05) | -0.00 (0.05) | -0.00 (0.05) |
| Acting with awareness |  | -0.85^***^ (0.16) | -0.84^***^ (0.16) |
| Solitude × acting with awareness |  |  | -0.06 (0.08) |
| ICC | 0.47 | 0.43 | 0.43 |
| Residual variance | 2.33 | 2.33 | 2.33 |
| Model fit (*R^2^*) | 0.05 | 0.11 | 0.11 |
| *LAN affect:* ***nonjudging of inner experiences*** *as the moderator* |  |  |  |
| Intercept | 3.52^***^ (0.15) | 3.51^***^ (0.15) | 3.51^***^ (0.15) |
| Age | -0.02^**^ (0.01) | -0.02^*^ (0.01) | -0.02^*^ (0.01) |
| Education level | 0.29 (0.24) | 0.28 (0.24) | 0.28 (0.24) |
| Marital status | -0.42 (0.34) | -0.29 (0.33) | -0.29 (0.33) |
| Living status | -0.30 (0.44) | -0.38 (0.43) | -0.39 (0.43) |
| Solitude | -0.00 (0.05) | -0.00 (0.05) | -0.00 (0.05) |
| Nonjudging of inner experiences |  | -0.58^***^ (0.17) | -0.56^***^ (0.17) |
| Solitude × nonjudging of inner experiences |  |  | -0.05 (0.08) |
| ICC | 0.47 | 0.45 | 0.45 |
| Residual variance | 2.33 | 2.33 | 2.33 |
| Model fit (*R^2^*) | 0.05 | 0.08 | 0.08 |

*Note*. *N* = 188. SE = Standard error; ICC = intraclass correlation. ^*^*p* < 0.05. ^**^*p* < 0.01. ^***^*p* < 0.001.
